# Fetal Alcohol Spectrum Disorders: Experimental Treatments and Strategies for Intervention

**Published:** 2011

**Authors:** Nirelia M. Idrus, Jennifer D. Thomas

**Keywords:** Fetal alcohol spectrum disorders, prenatal alcohol exposure, pregnancy, teratogenic effects, treatment, treatment models, experimental treatments, interventions, nutritional interventions, pharmacological interventions, environmental interventions

## Abstract

Despite the known damaging effects of prenatal alcohol exposure, women continue to drink during pregnancy, creating a need for effective interventions and treatments for fetal alcohol spectrum disorders (FASD). Experimental models can be useful in identifying potential treatments, and this article describes the spectrum of experimental therapeutics that currently are being investigated, including pharmacological, nutritional, and environmental/behavioral interventions. Some treatments target the underlying mechanisms that contribute to alcohol-induced damage, protecting against alcohol’s teratogenic effects, whereas other treatments may enhance central nervous system plasticity either during alcohol exposure or long after alcohol exposure has ceased. The insights gained to date from experimental models offer several candidates for attenuating the deficits associated with FASD.

Alcohol consumption during pregnancy is the most common preventable cause of birth defects in the Western world, producing a range of physical, neurological, and behavioral alterations known as fetal alcohol spectrum disorders (FASD). Although warning labels and other public health messages have been issued, some pregnant women and women of child-bearing age continue to drink (Centers for Disease Control and Prevention 2009), making it necessary to seek out treatments that might minimize or attenuate FASD. Identifying effective interventions is among the top priorities of FASD research.

An increasing number of clinical studies are examining potential behavioral and educational interventions for FASD (see the article by Paley and O’Connor in this issue, pp. 64–75). Basic science models also have been useful in identifying treatments that may be translated to clinical practice. When investigating potential treatments, researchers can choose several strategies. One strategy is to identify the numerous mechanisms that contribute to alcohol’s teratogenic effects and to block or prevent those actions. A second approach is to identify protective and provocative factors that may be controlled to improve outcomes. For example, maternal alcoholism may lead to malnutrition, which can exacerbate alcohol’s damaging effects, suggesting that nutritional interventions might reduce FASD. Another approach is to use what is known of factors that have beneficial effects on development and/or to enhance neuronal plasticity, even after the alcohol-induced damage is complete. This article will summarize the latest experimental therapeutics currently being investigated. Although identified primarily in animal models of developmental alcohol exposure, these treatments show promise in attenuating some FASD.

## Interventions During Alcohol Exposure

Alcohol disrupts development through numerous mechanisms, both direct and indirect. Several investigators have attempted to prevent alcohol-induced damage by blocking these mechanisms, as discussed below.

## *N*-Methyl-d-Aspartate Receptor Antagonists

**Figure 1:**
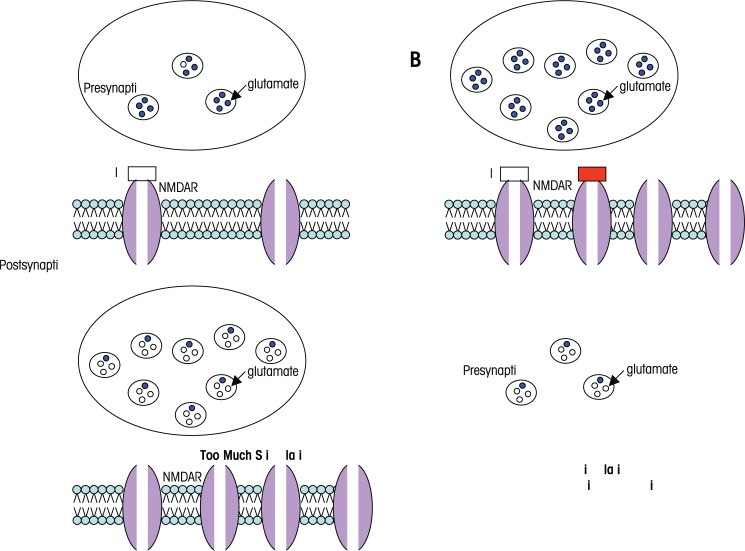
*N*-methyl-d-aspartate receptor (NMDAR)-mediated excitotoxic cell death. During alcohol withdrawal, it is postulated that overactivity of the NMDAR leads to excitotoxic cell death in the developing brain. **A)** Alcohol directly interacts with the NMDA receptor, which is activated by glutamate. Acute alcohol exposure inhibits the receptor, which contributes to the sedative and intoxicating effects of alcohol. **B)** Continued alcohol exposure may produce an adaptive neurocompensatory response, either as an increase in the number of NMDARs or an increase in the amount of glutamate released, which contributes to acute tolerance to alcohol’s intoxicating effects. However, when alcohol is eliminated from the body during periods of withdrawal, there may be rebound overactivity of NMDARs **(C)**, which may lead to cell death **(D)**.

Both animal and clinical studies report that binge drinking is associated with an increased risk of FASD. Binge drinking may be more damaging because it leads to higher blood alcohol concentrations and is linked to increased episodes of alcohol withdrawal. Alcohol interacts with a number of neuronal membrane binding molecules (i.e., receptors), including the *N*-methyl-d-aspartate (NMDA) receptor, which normally is activated by the excitatory brain chemical (i.e., neurotransmitter) glutamate. The NMDA receptor plays an important role in neuronal plasticity during development and later in life during learning. However, if it becomes overactive it can lead to increases in intracellular calcium and consequent cell death, a process called excitotoxicity. Alcohol acutely blocks the NMDA receptor, but chronic exposure may lead to a compensatory increase in either receptor number or glutamate release, actions that may contribute to tolerance (Crews et al. 1996; Grant and Lovinger 1995; Lovinger 1993). However, when alcohol is eliminated during withdrawal periods, it is postulated that overactivity of the NMDA receptor leads to excitotoxic cell death. (Tsai and Coyle 1998). This process also may occur in the developing brain (see figure 1).

**Figure 2:**
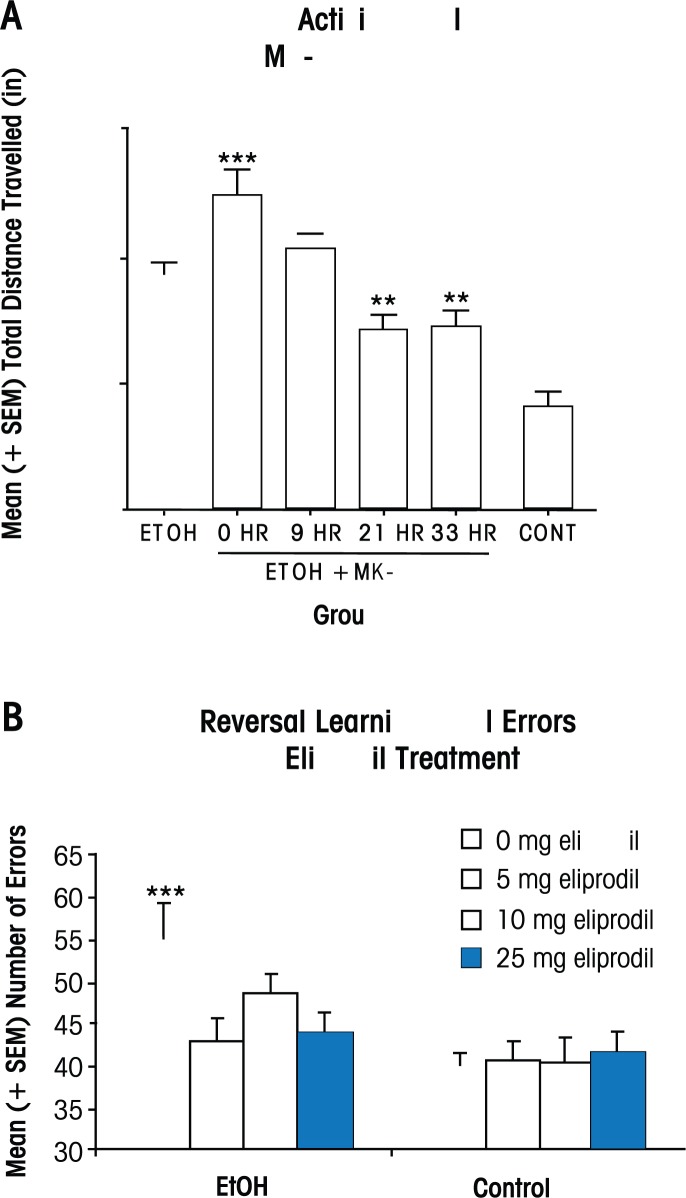
*N*-methyl-d-aspartate receptor (NMDAR) antagonists administered during alcohol withdrawal can attenuate some alcohol effects. **A)** Administration of the NMDAR antagonist MK-801 reduces the severity of alcohol-related open-field hyperactivity (Thomas et al. 2001) but only when administered during the withdrawal phase (21 and 33 hours after alcohol exposure). Note that the activity levels, even of those treated with MK-801 during the withdrawal period, do not reach those of the control subjects. **B)** Administration of the NMDAR antagonist eliprodil during the withdrawal period attenuated deficits on a spatial discrimination reversal learning task. Animals exposed to alcohol committed a significantly greater number of errors compared with all other groups. Animals exposed to alcohol and then treated with eliprodil during withdrawal committed significantly fewer errors compared with the alcohol group, reaching levels comparable with the controls (Thomas et al. 2004). Notes: EtOH = ethanol (i.e., alcohol)-exposed subjects; CONT/Control = control subjects; *** = significantly different from all other groups; ** = significantly different from all other groups except EtOH + MK-801 21 or 33 group.

Consistent with this mechanism, administration of NMDA receptor antagonists, drugs that block the NMDA receptor (e.g., MK-801, agmatine, eliprodil, memantine) during the withdrawal period, can attenuate some of alcohol’s teratogenic effects. For example, Thomas and colleagues (2001) demonstrated that MK-801 administration during alcohol withdrawal in developing rats reduces the severity of alcohol-related hyperactivity observed in an open field, improves the animals’ ability to react to changing contingencies (i.e., reversal learning), and protects against alcohol-induced hippocampal cell loss, but only when administered during the withdrawal phase and at low doses (Thomas et al. 2001) (see figure 2A). When administered at the same time as alcohol, MK-801 is highly toxic and exacerbates alcohol’s effects (Thomas et al. 2001). Fortunately, there are other NMDA receptor antagonists that may be safer than the potent MK-801. Drugs that act on the polyamine modulatory site of the NMDA receptor (e.g., eliprodil, agmatine) have attenuated deficits on a spatial discrimination reversal learning task (Thomas et al. 2004*a*) (see figure 2B) and motor balance deficits associated with early neonatal alcohol exposure (Lewis et al. 2007). More recently, Idrus and colleagues (2011) demonstrated that memantine, a drug believed to block NMDA receptor overactivation without blocking normal neurotransmission, mitigates alcohol-related motor coordination deficits, with concomitant neuroprotection against alcohol-induced cerebellar neuronal loss. Similarly, in vitro studies indicate that memantine is neuroprotective against hippocampal cell loss associated with developmental alcohol exposure (Stepanyan et al. 2008). These findings suggest that blocking NMDA receptors during withdrawal in the fetus or newborn may protect against some of the neuropathology associated with prenatal alcohol exposure.

### Serotonin Agonists

Alcohol also acts on other neurotransmitter systems, including the serotonergic (5-hydroxytryptamine [5-HT]) systems (see Valenzuela and colleagues, pp. 106–120, in this issue). Developmental alcohol exposure decreases both 5-HT neuronal number and serotonin levels. Importantly, not only does serotonin act as a neurotransmitter but it also promotes neuronal growth (i.e., as a neurotrophic factor, a substance that supports and maintains neurons). Druse and colleagues (2004) have shown that administration of serotonin agonists (e.g., ipsapirone, busipirone) either in vivo or in vitro can prevent alcohol-induced cell death (i.e., apoptosis) and loss of neurons and glia within brain regions that contain 5-HT cells (Druse et al. 2004; Zhou et al. 2008), possibly by increasing anti-apoptotic proteins (Lee et al. 2009).

### Antagonism of Alcohol Effects on L1 Cell Adhesion Molecules

Alcohol also may interfere with normal neurodevelopment by reducing cell adhesion. Such cell-to-cell contact is imperative for neuronal communication, influencing neuronal growth, development and survival, and, in particular, cellular migration and the development of axons and dendrites. Alcohol’s ability to specifically inhibit the adhesive properties and axon and dendrite outgrowth mediated by the L1 cell adhesion molecule (CAM) has implicated L1CAM as a target for developmental alcohol neurotoxicity (Bearer 2001).

**Figure 3:**
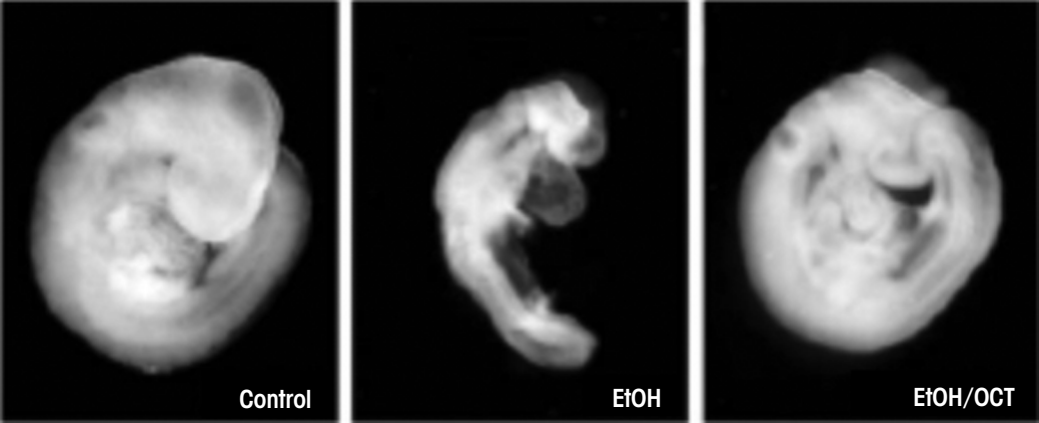
Disrupting alcohol’s effects on L1 cell adhesion molecules reduces teratogenic effects. Ethanol (EtOH) (i.e., alcohol) can specifically inhibit the adhesive properties and development of axons and dendrites mediated by the L1–cell adhesion molecule (L1CAM). 1-octanol (OCT) can prevent alcohol-related teratogenic effects. Compared with the control mouse embryo, an alcohol-exposed embryo exhibits severe alterations in development. Administration of 1-octanol during alcohol exposure, which prevents alcohol from inhibiting L1CAM, attenuates these alterations. SOURCE: Reprinted with permission from Chen, S.Y.; Wilkemeyer, M.F.; Sulik, K.K.; and Charness, M.E. Octanol antagonism of ethanol teratogenesis. *FASEB Journal* 15:1649–1651, 2001a. PMID: 11427515

Because of their capacity to disrupt ethanol’s action on L1 adhesion, 1-octanol and other alcohols can prevent ethanol-induced apoptotic cell death, growth retardation, and neural tube closure delays (Chen et al. 2001*a*) (see figure 3). These findings suggest that development of pharmacological agents that prevent alcohol-induced inhibition of L1 cell adhesion may serve as therapeutic agents to reduce the severity of FASD. The potential development of such agents recently has been enhanced by the identification of the alcohol-binding site on the L1CAM (Arevalo et al. 2008).

In addition, peptide fragments derived from neurotrophic factors like glial-derived activity-dependent neuroprotective protein (ADNP) and activity-dependent neurotrophic factor (ADNF) also can reduce alcohol’s teratogenic effects, in part by antagonizing alcohol’s inhibition of L1 cell adhesion (Wilkemeyer et al. 2002). The active peptide fragments, NAPVSIPQ (NAP) and SALLRSIPA (SAL), which are derived from ADNP and ADNF, respectively, have demonstrated strong neuroprotection, preventing alcohol-related fetal death, physical alterations such as neural tube and ocular defects, reductions in brain weight and volume, neuronal cell loss including the loss of serotonergic neurons, and even spatial learning deficits. Although NAP and SAL can protect against a variety of neural insults, the ability of NAP to protect against alcohol’s teratogenesis is related to its ability to antagonize alcohol’s effects on L1 cell adhesion. Peptide derivatives of NAP that lack neuroprotective properties against other insults still can prevent alcohol teratogenesis, whereas derivatives that weakly block alcohol’s effects on L1 remain neuroprotective against other insults but are less effective in blocking alcohol teratogenesis (Wilkemeyer et al. 2003).

### Neurotrophic Factors

Neurotrophic factors influence cell metabolism and growth, proliferation, differentiation, migration and maturation of cells, and apoptotic cell death. Alcohol exposure during development may impair neurotrophic factor production. In addition, when immature neurons are exposed to alcohol, the newly developed cells may respond abnormally to certain guidance or trophic factors (Lindsley et al. 2003). Alteration by alcohol to either of these mechanisms may lead to a pathological cascade of events, which may ultimately contribute to FASD.

In the presence of alcohol, the levels of these neurotrophic factors, including insulin-like growth factor, nerve growth factor, basic fibroblast growth factor, brain-derived neurotrophic factor, and glial-derived neurotrophic factor, are reduced (e.g., Climent et al. 2002). Conversely, as noted with NAP and SAL above, the administration of many of these growth factors can ameliorate alcohol’s teratogenic effects (e.g., Mitchell et al. 1999). Such effects can range from mitigating alcohol-induced motor deficits (McGough et al. 2009) to increasing rates of survival for neurons and their axons and dendrites (Barclay et al. 2005) (see table). Such a broad range of mitigative effects may well be attributed to the large overarching contributions of neurotrophic factors to the development of the CNS.

These findings highlight the potential of neurotrophic factors as possible treatments for FASD. It also is important to note that neurotrophic factors may reduce alcohol’s teratogenic effects by promoting cell growth and survival, even if alcohol does not directly affect that neurotrophic system. One challenge with in vivo administration of neurotrophic factors is protecting against alcohol’s teratogenic effects without disrupting normal developmental events.

### Antioxidants

**Figure 4:**
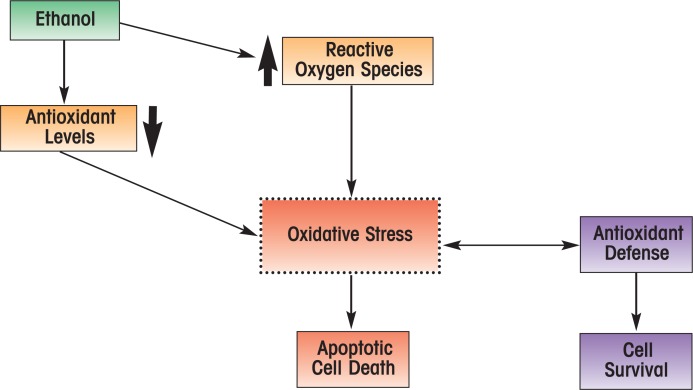
Ethanol (i.e., alcohol) has numerous actions on the developing organism, including a reduction in the levels of antioxidants and an increase in reactive oxygen species, which creates a state of oxidative stress. This can lead to extensive apoptotic cell death. However, the administration of a range of antioxidants can mitigate many of alcohol’s teratogenic effects, including growth retardation, physical anomalies, and neuronal loss.

Alcohol also may lead to cell death via an imbalance between the production of damaging reactive oxygen species and the ability of cells to protect themselves with endogenous antioxidants (i.e., oxidative stress) (see figure 4). Developmental alcohol exposure induces extensive apoptotic cell death, which is preceded by an increase in reactive oxygen species and low levels of protective antioxidants (Ramachandran et al. 2003). Successful protection against alcohol-related growth retardation, physical anomalies, and neuropathologies has been illustrated with a number of antioxidant agents, including resveratrol from red wine, curcumin from tumeric, and epigallocatechin-3-gallate from green tea (Antonio and Druse 2008). Similarly, the superoxide dismutase/catalase mimetic EUK-134 (Chen et al. 2004), and even the induction of Nrf2 protein, which activates endogenous antioxidant enzymes (Dong et al. 2008), can block alcohol’s teratogenic effects. Many studies have illustrated similar beneficial effects with the antioxidant vitamins E, C, and beta carotene (a form of vitamin A) (Cohen-Kerem and Koren 2003). Even chemically engineered forms of vitamin E, designed for the accumulation of vitamin E within the mitochondria, have shown promise, albeit only in in vitro studies thus far (Siler-Marsiglio et al. 2005). However, to date, the behavioral consequences of antioxidants are equivocal, and not all studies find beneficial effects. Moreover, a clinical study examining megadose supplementation of vitamins C and E in women who were drinking during pregnancy was terminated after reports that high levels of vitamin C and E may lead to low birth weight among women with pre-eclampsia (Goh et al. 2007). Nevertheless, given the large number of antioxidant agents and methods for manipulating antioxidant machinery, identification of antioxidants that could be effective clinically still is of great interest.

### Nutritional Factors

As noted above, vitamins C and E possess antioxidant properties. Other nutrients also may influence alcohol’s teratogenic effects. Outcomes among children exposed to alcohol during pregnancy vary widely, and some of this variation may be attributed to nutritional factors. For instance, higher rates of FASD are observed in countries where malnutrition is prevalent (May et al. 2000). It therefore is likely that malnutrition can worsen fetal alcohol effects. In fact, alcohol is known to interfere with nutritional supply to the unborn fetus. In contrast, nutritional supplements may attenuate alcohol’s adverse effects on fetal development.

For example, zinc deficiency may contribute to some FASD. Although zinc supplementation in one study did not mitigate alcohol-induced cerebellar Purkinje cell loss following developmental alcohol exposure (Chen et al. 2001*b*), the administration of zinc has been shown to protect against postnatal mortality, fetal dysmorphology, and cognitive impairments in the offspring of alcohol-treated dams (Summers et al. 2008, 2009). Similarly, folic acid deficiency during pregnancy is well known to induce neural tube defects, and folate supplementation can help protect against these congenital malformations. Prenatal folic acid supplementation mitigates many of alcohol’s teratogenic effects, including growth retardation, physical anomalies, and neuronal loss (Wang et al. 2009).

Administration of another nutrient, nicotinamide (one of the B complex vitamins), during or shortly after developmental alcohol exposure in mice, also protects against alcohol-related apoptotic cell death, as well as contextual fear conditioning deficits and overactivity in the open field (Ieraci and Herrera 2006). Importantly, nicotinamide did not alter the pharmacokinetics of alcohol. However, it should be noted that the doses of nicotinamide used in this study translate to much higher doses than those used in clinical populations. Thus, it is not clear if nicotinamide’s actions are functioning at a nutritional or pharmacological level. Nevertheless, the findings are intriguing.

Thomas and colleagues (2009, 2010) also have reported that prenatal choline supplementation can mitigate alcohol’s teratogenic effects, decreasing the severity of alcohol-related birth weight reductions, physical anomalies, and alterations in behavioral development. Choline is an essential nutrient that may mitigate alcohol’s teratogenic effects via various mechanisms, acting as a methyl donor, as a precursor to components of the cell membranes, or as a precursor to the neurotransmitter acetylcholine.

Given the known damaging effects of nutritional deficiencies on the developing fetus, nutritional supplements may serve as a relatively easy means to improve outcomes among alcohol-exposed offspring. However, one must keep in mind that there are synergistic effects among nutrients. For example, iron deficiency can exacerbate prenatal alcohol effects on growth (Carter et al. 2007), and although iron supplementation could have mitigating effects on FASD, it also can impair zinc absorption. Therefore, a balanced multisupplement diet throughout pregnancy may be most effective. Finally, it is important to note that nutritional interventions may be effective even if alcohol is not inducing nutritional deficiencies; in other words, nutritional interventions may be effective whether or not they are compensating for a deficiency. Certainly, further investigations of nutritional interventions for reducing FASD are needed.

## Treatments for Individuals With FASD

For the most part, the experimental treatments described above must be given during fetal development, during the alcohol insult, to be effective. For example, administration of antioxidants must occur during oxidative stress, and the administration of NMDA receptor antagonists is only effective when given during alcohol withdrawal. In a clinical setting, at-risk populations could be targeted for such therapeutic interventions. However, it is difficult to identify at-risk women and ensure treatment compliance. Prenatal treatment(s) may therefore be difficult to implement. Instead, focusing interventions on alcohol-affected children may be much more clinically relevant. Indeed, some of the treatments that are effective during the alcohol insult still may be effective when administered after alcohol exposure. For example, some neurotrophic factors can enhance neuronal survival and synaptic connections, and can improve behavioral outcomes even when administered after alcohol exposure (e.g., McGough et al. 2009).

### Nutritional Supplementation

**Figure 5:**
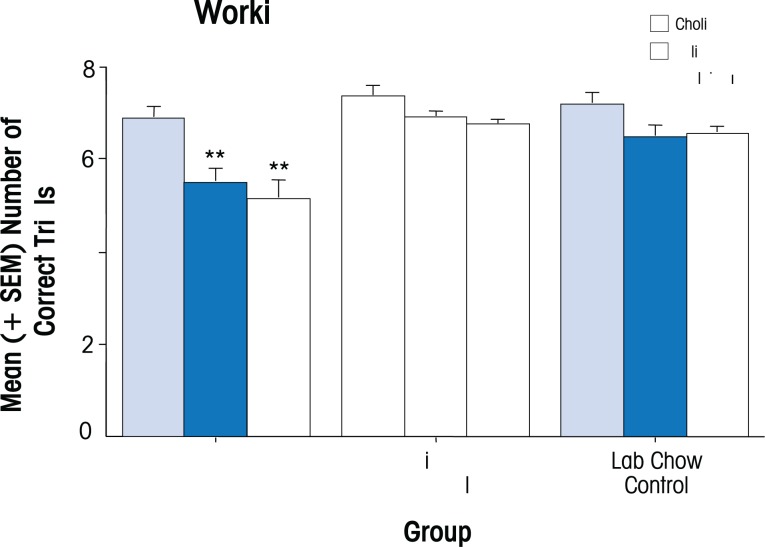
Choline supplementation during early postnatal development mitigates cognitive deficits induced by prenatal ethanol (i.e., alcohol) exposure. Prenatal alcohol exposure significantly impaired performance on a working memory learning task among adult rats. When the alcohol-exposed animals received choline during early postnatal development, well after alcohol exposure, this effect was mitigated. Notes: EtOH = alcohol-exposed animals; ** = significantly different from all other groups SOURCE: Reprinted with permission from Thomas, J.D.; La Fiette, M.H.; Quinn, V.R.; and Riley, E.P. Neonatal choline supplementation ameliorates the effects of prenatal alcohol exposure on a discrimination learning task in rats. *Neurotoxicology and Teratology* 22:703–711, 2000. PMID: 11106863

As mentioned above, administration of choline during prenatal alcohol exposure can reduce the severity of adverse physical and behavioral outcomes. Choline also may be effective even when administered after the alcohol exposure has ceased and during periods of postnatal development. The first study to show the beneficial effects of choline following developmental alcohol exposure administered choline for 3 weeks following birth in rats exposed to alcohol during gestation. Choline reduced the severity of alcohol-related working memory deficits observed in adulthood, illustrating that choline’s effects were long lasting (Thomas et al. 2000) (see figure 5). Postnatal choline supplementation (up to postnatal day 30) also can reduce the severity of overactivity in the open field, reversal learning deficits, spatial learning deficits, and impairments in trace classical conditioning (i.e., learning conditioned responses to eye blink-eliciting stimuli and fear-eliciting stimuli) associated with developmental alcohol exposure (Thomas et al. 2004*b*; Wagner and Hunt 2006). More recently, Ryan and colleagues (2008) demonstrated that administration of choline from either postnatal days 11–20 or 21–30 could improve behavioral outcome. In fact, choline supplementation during adolescence/young adulthood in the rat (postnatal day 40–60) can improve cognitive performance. Notably, choline loading during this later developmental period did not improve simple spatial learning or normalize activity levels in the open field but did significantly reduce the severity of alcohol-related working memory deficits. These findings suggest that choline administration later in development may target the prefrontal cortex. Importantly, in all of these studies, behavioral testing occurred after choline treatment, so benefits were not related to acute effects of choline. These data hold promise that nutritional interventions may be effective among individuals with FASD. Researchers currently are investigating the changes in brain functioning following perinatal choline supplementation and the mechanisms of choline’s actions.

### Pharmacological Interventions

CNS plasticity (the ability of the brain to change) continues throughout the lifespan. Although CNS plasticity can be compromised following developmental alcohol exposure, the use of agents that enhance plasticity still may be effective. For example, a developmental alcohol insult will impair the activation of cAMP response element–binding protein (CREB) (Krahe et al. 2009), which is necessary for memory formation and retrieval, and other components of neuronal plasticity. Administration of agents that inhibit the enzyme phosphodiesterase (PDE) can prolong CREB activation and facilitate long-term potentiation, a strengthening of synaptic connections that may contribute to learning and memory. In addition, a study in developing ferrets found that PDE inhibitors administered 1 week after an alcohol insult increased CREB activation and restored sensory cortical plasticity (Krahe et al. 2009; Medina et al. 2006). Thus, even though alcohol may impair baseline plasticity, it may be restored with appropriate interventions. Targeting plasticity in such a manner may be particularly effective when measures to prevent drinking during pregnancy fail.

### Environmental Interventions

Neuronal plasticity also can be enhanced with environmental enrichment. A typical enriched environment will include social, motor, and sensory stimulation using running wheels and toys, items that the animals can crawl into, play on, manipulate, and chew. Communal rearing, neonatal handling, and exercise also can act as enriched environmental factors. Stimulation using such enrichment programs is known to elicit a number of CNS responses, ranging from increases in neurotrophic factor levels to structural changes including dendritic arborization and neurogenesis to improved learning. A variety of environmental enrichment paradigms have been shown to improve behavioral outcomes, including motor, social, and cognitive functioning, following prenatal alcohol exposure (see Hannigan et al. 2007). For example, animals exposed to alcohol in utero and then reared in standard cages exhibit abnormal gaits. When alcohol-exposed animals were reared in an enriched environment, their gait did not differ from that of controls (Hannigan et al. 1993). Similarly, exercise on running wheels can attenuate the adverse effects of developmental alcohol exposure on hippocampal plasticity and learning (Christie et al. 2005).

Certain brain regions also can be targeted using specific enriched environment paradigms. For example, acrobatic motor learning can enhance functioning of the cerebellum and attenuate alcohol-induced motor skill impairments (Klintsova et al. 2000). Although animals exposed to alcohol during development initially were not as skilled as the controls, after days of training, they eventually were able to perform at control levels. Even though significant cerebellar Purkinje cell loss remained following early alcohol exposure, an increase in the number of synaptic connections for each of the remaining Purkinje cells was found, showing the capacity for synaptic plasticity (Klintsova et al. 2002). Such mitigative effects are persistent and can be observed weeks after environmental enrichment.

In contrast, some studies have shown that brains exposed to alcohol during development do not possess the same capacity for plasticity as non–alcohol-exposed brains. For example, one study failed to find an increase in adult hippocampal neurogenesis following environmental enrichment in mice prenatally exposed to alcohol. In comparison, control animals reared in the enriched environment showed a twofold increase in neurogenesis (Choi et al. 2005). Thus, it is important to determine how much plasticity is maintained by the alcohol-exposed subject and what manipulations will be most effective in capitalizing on existing plasticity. Moreover, in addition to identifying experiences that can improve outcome, one may need to identify and protect against adverse experiences, such as early stress, that may exacerbate alcohol’s teratogenic effects (see Abel and Hannigan 1995). Clearly, the postnatal environment can be key in influencing the adverse effects of developmental alcohol exposure.

## Conclusions

Individuals born with FASD suffer from a lifetime of physical, cognitive, and behavioral problems. Ideally, one would intervene at the time of alcohol exposure, directly preventing or reducing maternal alcohol consumption. However, because prevention is not always possible, there is a need to seek effective treatments that will mitigate developmental alcohol-related deficits. The insights gained to date from experimental models offer several candidates for the attenuation of deficits associated with prenatal alcohol exposure. Although translating these treatments to the clinical arena poses challenges, including the difficulty of administering treatments during prenatal periods as well as safety concerns, continued research and refinement may lead to viable clinical treatments. Moreover, some treatments, including nutritional, behavioral, and environmental manipulations, can be much more readily translated. Because some women around the world continue to drink alcohol during pregnancy, it is critical to continue pursuing effective interventions. The present findings suggest that various treatments have the potential to attenuate alcohol’s adverse effects and improve the quality of life of individuals with FASD.

## Figures and Tables

**Table:** Neurotrophic factors are a family of proteins that can mitigate a range of alcohol-related disruptions in development, increasing cell survival, improving the regulation of axonal and dendritic processes, and ameliorating behavioral deficits. This Table provides examples of these actions.

**Neurotrophic Factors**	**Properties**	**Influence on Fetal Alcohol Effects**
Insulin-like Growth Factor	Effects on cell metabolism and growth; promotes proliferation, differentiation, and maturation of neurons and glia; reduces apoptosis	Significantly mitigated motor impairments in vivo; increased neuronal survival in vitro; and promotes neuronal viability in vitro (e.g., McGough et al. 2009)
Nerve Growth Factor	Critical for the survival and maintenance of sympathetic and sensory neurons	Increased total dendrite and axon length in vitro; and increased neuronal survival in vitro (e.g., Mitchell et al. 1999)
Basic Fibroblast Growth Factor	Mediates the formation of angiogenesis (blood vessels) and promotes neuronal survival	Increased neuronal survival in vitro (e.g., Barclay et al. 2005)
Brain Derived Neurotrophic Factor	Aids in neuronal survival, encourages growth and differentiation of new neurons and synapses	Promoted neuronal survival and viability in vitro (e.g., Climent et al. 2002)
Glial-Derived Neurotrophic Factor	Promotes survival and differentiation of many neuronal types; may prevent apoptotic cell death	Increased neuronal survival in vitro (e.g., McAlhany et al. 1997)
